# All-Optical Encryption and Decryption at 120 Gb/s Using Carrier Reservoir Semiconductor Optical Amplifier-Based Mach–Zehnder Interferometers

**DOI:** 10.3390/mi16070834

**Published:** 2025-07-21

**Authors:** Amer Kotb, Kyriakos E. Zoiros, Wei Chen

**Affiliations:** 1 School of Chips, XJTLU Entrepreneur College (Taicang), Xi’an Jiaotong-Liverpool University, Suzhou 215400, China; 2 Department of Physics, Faculty of Science, University of Fayoum, Fayoum 63514, Egypt; 3 Lightwave Communications Research Group, Department of Electrical and Computer Engineering, School of Engineering, Democritus University of Thrace, 67100 Xanthi, Greece; kzoiros@ee.duth.gr

**Keywords:** all-optical encryption/decryption, XOR logic gate, carrier reservoir semiconductor optical amplifier, Mach–Zehnder interferometer, quality factor

## Abstract

Encryption and decryption are essential components in signal processing and optical communication systems, providing data confidentiality, integrity, and secure high-speed transmission. We present a novel design and simulation of an all-optical encryption and decryption system operating at 120 Gb/s using carrier reservoir semiconductor optical amplifiers (CR-SOAs) embedded in Mach–Zehnder interferometers (MZIs). The architecture relies on two consecutive exclusive-OR (XOR) logic gates, implemented through phase-sensitive interference in the CR-SOA-MZI structure. The first XOR gate performs encryption by combining the input data signal with a secure optical key, while the second gate decrypts the encoded signal using the same key. The fast gain recovery and efficient carrier dynamics of CR-SOAs enable a high-speed, low-latency operation suitable for modern photonic networks. The system is modeled and simulated using Mathematica Wolfram, and the output quality factors of the encrypted and decrypted signals are found to be 28.57 and 14.48, respectively, confirming excellent signal integrity and logic performance. The influence of key operating parameters, including the impact of amplified spontaneous emission noise, on system behavior is also examined. This work highlights the potential of CR-SOA-MZI-based designs for scalable, ultrafast, and energy-efficient all-optical security applications.

## 1. Introduction

In the era of ever-increasing data traffic and the proliferation of high-speed communication networks, the demand for ultrafast, secure, and energy-efficient information processing systems is more critical than ever. Among the many strategies developed to meet these demands, all-optical encryption and decryption schemes have gained significant traction. Unlike traditional electronic encryption systems that require optical-electrical-optical (OEO) conversions—thereby introducing latency, energy consumption, and bottlenecks—all-optical encryption and decryption systems operate entirely in the optical domain. This enables data to be processed at the speed of light, with minimal latency and superior bandwidth handling capabilities. These systems provide data confidentiality and integrity directly in the photonic layer, which is crucial for next-generation secure communication infrastructures, especially in military, banking, quantum communication, and cloud-based platforms [1,2,3,4,5,6,7,8]. All-optical encryption typically employs binary logic operations, where the input signal (plaintext) is combined with a secure key using a logic function—most commonly the exclusive OR (XOR) logic gate—to generate a scrambled output (ciphertext). Decryption is then achieved by repeating the XOR operation with the same key, restoring the original data. This simplicity and reversibility make the XOR logic gate an ideal candidate for optical encryption and decryption applications [1,2,3,4,5]. Beyond encryption, all-optical XOR gates play a vital role in optical computing, signal regeneration, pattern recognition, error detection/correction, and arithmetic operations [9,10]. Their implementation using nonlinear optical elements allows ultrafast logic processing directly in the optical domain, a key enabler for all-optical networks and photonic integrated circuits.

Among the various optical devices used to realize such functions, semiconductor optical amplifiers (SOAs) have been widely studied due to their compactness, integrability, low power consumption, and strong nonlinear characteristics such as cross-phase modulation (XPM) and cross-gain modulation (XGM). However, a major physical limitation of conventional SOAs is their relatively slow carrier recovery time, typically on the order of hundreds of picoseconds. This restricts their ability to operate effectively at very high bit rates, particularly beyond 100 Gb/s, due to inter-symbol interference, gain saturation, and pattern-dependent distortion [11]. These issues make it challenging to maintain signal fidelity and clear logic states in high-speed optical logic circuits, thereby necessitating the development of enhanced SOA structures such as carrier reservoir semiconductor optical amplifiers (CR-SOAs). CR-SOAs introduce an additional carrier reservoir region that decouples the carrier injection from the signal interaction region. This design enables faster gain recovery times, improved saturation characteristics, and better control over carrier dynamics, making them particularly suitable for high-speed and high-fidelity all-optical logic operations [12,13,14,15,16,17,18]. These characteristics also reduce pattern-dependent effects and crosstalk, both of which are critical in logic-based encryption systems operating at elevated bit rates.

To implement reliable all-optical logic gates, one of the most widely adopted configurations is the Mach–Zehnder interferometer (MZI). When integrated with nonlinear optical elements like SOAs or CR-SOAs in its arms, the MZI can perform high-speed logic functions by leveraging XPM or XGM. The phase-sensitive interference at the MZI output allows for precise control of logic outcomes, such as XOR, with high extinction ratios and low insertion losses. MZIs offer significant advantages in terms of reconfigurability, stability, and scalability, making them an essential building block in integrated photonic circuits and optical logic systems [19,20]. The combination of CR-SOAs and MZIs creates a powerful platform for realizing compact, high-speed, and energy-efficient logic devices suitable for data security applications.

In recent years, various theoretical and experimental demonstrations of all-optical encryption and decryption systems based on SOAs have been reported in the literature [1,2,3,4,5,6,7,8]. These studies span designs using both traditional SOAs [1,2,3,4,5,6] and enhanced variants like quantum-dot SOAs (QD-SOAs) [7] and reflective SOAs (RSOAs) [8]. While some employ complex configurations involving pseudorandom bit generators, linear feedback shift registers, and two-photon absorption (TPA) mechanisms [6], others focus on more straightforward XOR-based schemes [1,2,3,4,5]. These works demonstrate the viability of SOA-based technologies for secure, real-time encryption, but also highlight challenges related to signal degradation, gain recovery, and noise issues that CR-SOAs are particularly well-suited to address.

An important metric in evaluating the performance of all-optical logic gates and encryption/decryption systems is the quality factor (QF). The QF serves as an indicator of signal integrity, noise tolerance, and eye diagram clarity in high-speed optical systems. A high QF corresponds to clean, distinguishable logical ‘1’ and ‘0’ states, which are essential for accurate data recovery and secure communication. In the context of encryption and decryption, a strong QF ensures that the ciphertext and the recovered plaintext maintain acceptable levels of fidelity, even in the presence of noise sources such as amplified spontaneous emission (ASE). Therefore, tracking the QF provides crucial insight into the practical feasibility and robustness of the proposed design.

In this work, we present a high-speed all-optical encryption and decryption system operating at 120 Gb/s, implemented using CR-SOAs-based MZIs and simulated in Wolfram Mathematica 11.0. The system utilizes two consecutive CR-SOA-MZI XOR gates to perform encryption and decryption, and achieves QFs of 28.57 and 14.48, respectively, for the encrypted and decrypted signals. Additionally, we analyze the impact of key operational parameters, including ASE noise, on system performance. The proposed design offers a promising pathway toward compact, ultrafast, and scalable photonic encryption systems, particularly suited for next-generation high-speed secure communication networks.

## 2. Principle of Operation

### 2.1. CR-SOA

**Figure 1:**
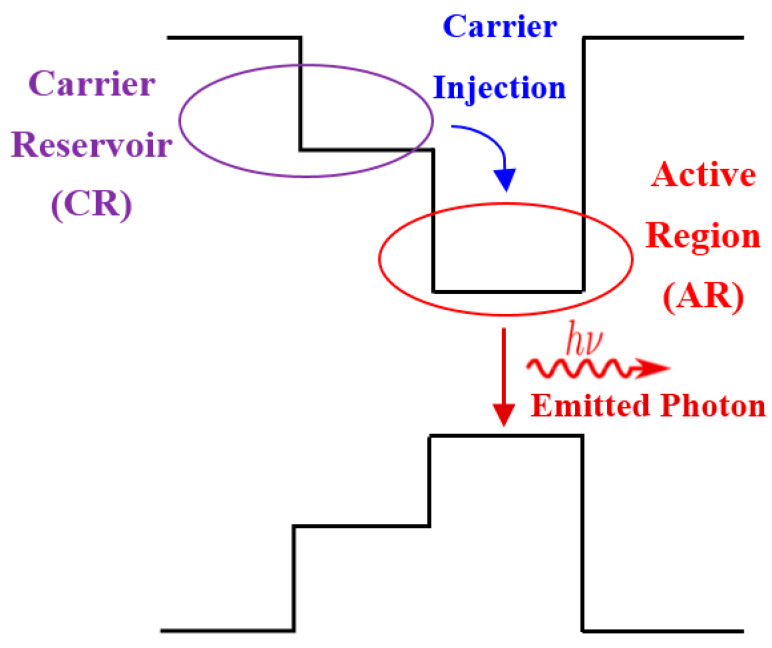
Schematic of the CR-SOA showing carrier injection, storage in the carrier reservoir (CR), transfer to the active region (AR), stimulated emission, and recombination.

CR-SOA is a modified version of the conventional SOA, designed to enhance dynamic performance and suppress nonlinear distortions in high-speed optical signal processing. It incorporates an additional carrier reservoir (CR) region alongside the active gain region (AR), where the CR serves as a carrier buffer that facilitates rapid replenishment during signal modulation. This structural modification decouples the carrier dynamics from the gain mechanism, resulting in faster carrier recovery, improved gain saturation, enhanced modulation bandwidth, and reduced pattern effects, making CR-SOAs suitable for all-optical logic gates and high-speed regeneration. Our simulation results confirm that CR-SOAs offer simpler integration and superior thermal stability compared to more complex nanostructured devices. While QD-SOAs exploit discrete energy states to achieve ultrafast carrier dynamics, low chirp, and high spectral purity, they often require more sophisticated fabrication and temperature control. However, recent advancements have improved the manufacturability of QD-SOAs, enabling their adoption in photonic neural networks and terabit optical transceivers. Nevertheless, for applications prioritizing integration simplicity, thermal robustness, and logic-level performance, CR-SOAs remain a highly practical and effective solution [11,12,13,14,15,16,17,18]. Figure 1 presents schematic diagrams illustrating the key carrier transitions within the CR-SOA, including injection, capture into the reservoir, and transfer to the active region, stimulated emission, and recombination.

### 2.2. XOR Gate

**Figure 2:**
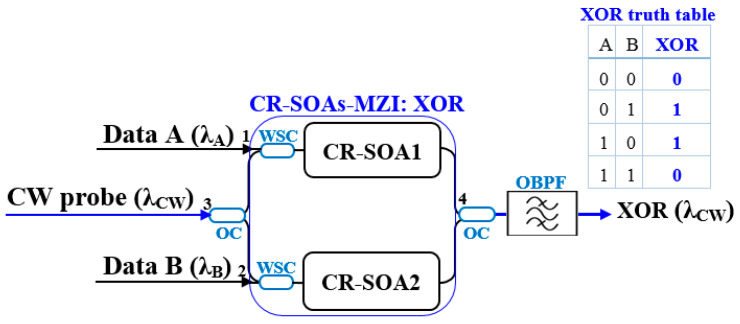
Diagram of the CR-SOA-MZI XOR gate with inputs via WSCs, CW split by a 3 dB OC, and output filtered by an OBPF. The inset shows the corresponding XOR truth table.

Figure 2 depicts the XOR gate implementation using CR-SOA-MZIs. The two input data signals, A and B, are launched into ports 1 and 2 through wavelength-selective couplers (WSCs). These WSCs serve to selectively route specific wavelength channels into the CR-SOA arms, enabling precise signal injection and isolation to minimize crosstalk. Simultaneously, a continuous-wave (CW) laser is injected into port 3 and then equally split into two identical optical beams by a 3 dB optical coupler (OC). This OC acts as a power splitter that divides the input CW beam into two coherent components with equal intensity, which travel through the two arms of the MZI. Within each arm, the CR-SOAs induce changes in the amplitude and phase of the CW beams through XGM and XPM, driven by the respective input signals A and B. When both inputs are identical (either ‘0’ or ‘1’), the gain and phase shifts applied to the two CW beams are matched, causing destructive interference at the output port and yielding a logic ‘0’. In contrast, when the inputs differ, the CW beams undergo distinct modulations, producing constructive interference and a logic ‘1’. An optical bandpass filter (OBPF) is used to remove unwanted spectral components and noise, ensuring a clean output signal that corresponds to the XOR truth table. This architecture has been experimentally validated in several studies [1,2,21], achieving high-speed XOR logic operation with excellent extinction ratios and low bit-error rates, confirming its practicality for ultrafast all-optical processing systems.

### 2.3. Encryption and Decryption

**Figure 3:**
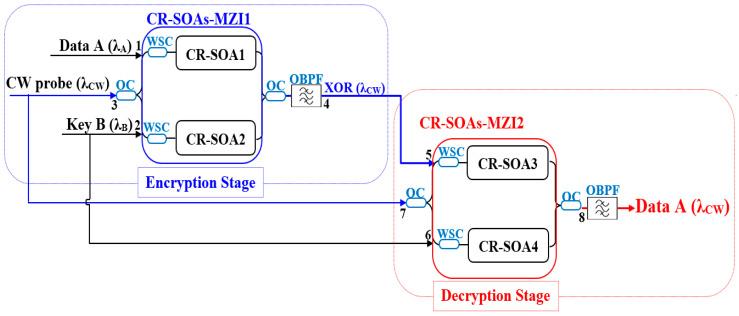
Schematic diagram of the all-optical encryption and decryption system using two cascaded CR-SOA-MZIs, showing the Encryption Stage (CR-SOA-MZI1) and Decryption Stage (CR-SOA-MZI2), with data and key signals processed via XOR operations.

The proposed all-optical encryption and decryption scheme employs two cascaded CR-SOA-MZIs, where signal A represents the input data and signal B acts as the encryption key. The logic is based on two successive XOR operations (A ⊕ B) to enable secure, reversible all-optical data processing, as shown in Figure 3. In the first stage, CR-SOA-MZI1 functions as the encryption module. Signals A and B are launched into its arms via WSCs, while a continuous wave (CW) probe at wavelength λ_CW_ is injected at port 3. A 3 dB optical coupler (OC) splits the CW equally between the two arms, where nonlinear interactions—cross-gain modulation (XGM) and cross-phase modulation (XPM)—modulate the CW depending on the input signals. Interference at the output port produces the encrypted signal, the XOR result (A ⊕ B). An optical bandpass filter (OBPF) is applied to suppress noise and unwanted spectral components, ensuring a clean encrypted output at λCW. In the second stage, CR-SOA-MZI2 serves as the decryption module. The encrypted signal (A ⊕ B) and the original key B, both now carried at λ_CW_, are introduced via WSCs and processed in a similar manner. The second XOR operation yields (A ⊕ B) ⊕ B = A, thus successfully retrieving the original data. This double-XOR mechanism using CR-SOA-MZI1 and CR-SOA-MZI2 provides a robust, high-speed framework for real-time optical encryption. The fast carrier recovery and reduced pattern dependence of CR-SOAs enable low-latency, scalable, and secure optical communication systems.

The truth table, as shown in Table 1, illustrates the functional behavior of the proposed all-optical encryption and decryption scheme using cascaded CR-SOA-MZIs, where signal A represents the input data and signal B acts as the encryption key. The encryption is performed in CR-SOA-MZI1 using the XOR operation A ⊕ B, and the decryption is carried out in CR-SOA-MZI2 by applying the same XOR logic again, yielding (A ⊕ B) ⊕ B = A. When A and B are identical, the encrypted output is ‘0’ and the decrypted result restores A as ‘0’; when they differ, the encrypted output becomes ‘1’ and the decrypted output accurately recovers A as ‘1’. This confirms the scheme′s logical correctness and ensures reliable, reversible optical data security.

## 3. Modeling

In this study, the QF is used as a key metric to evaluate the performance of the proposed optical logic functions. The QF provides insight into signal integrity by measuring the separation between logical ‘1’ and ‘0’ levels and their associated noise, which directly correlates with the bit error rate (BER) [22]—a critical parameter in high-speed communication systems. It is defined as [11]:

(1) $$QF=\frac{P_{1}-P_{0}}{\sigma_{1}+\sigma_{0}}$$

where P_1_ and P_0_ are the mean peak powers of logic levels ‘1’ and ‘0’, and σ_1_, σ_0_ represent their respective standard deviations. A QF value above 6 is typically required to maintain a BER below 10^−9^, ensuring system reliability [11,12,13,14,15]. To ensure precise results, all time-dependent equations are solved using the Adams numerical method implemented in Wolfram Mathematica^®^. Renowned for its efficiency in handling complex differential equations, this method is well suited for the detailed temporal analysis of optical systems. By incorporating the QF as the key performance metric and leveraging Mathematica’s accurate numerical capabilities, this study delivers a comprehensive assessment of the optical logic gate’s operation, capturing both overall system behavior and the influence of noise and distortion on signal integrity. The simulations are conducted using the standard numerical parameters outlined in Table 2 [1,2,3,4,5,6,7,8,9,10,11,12,13,14,15,16,17,18,19,20,21], which include experimentally validated values from prior studies [1,2,3], thereby ensuring consistency and robustness in the performance evaluation.

The dynamic behavior of the CR-SOA is governed by a set of time-dependent gain equations that accurately capture the complex interplay between carrier recombination processes and ultrafast nonlinear optical effects. Specifically, the model accounts for carrier exchange dynamics between the CR and the AR, which are fundamental to regulating the temporal evolution of the optical gain. Additionally, it includes the effects of carrier heating (CH) and spectral hole burning (SHB)—two key intraband phenomena that significantly influence both the amplitude and phase of the amplified optical signal. These ultrafast processes introduce gain suppression and nonlinear phase shifts that directly impact the performance of high-speed optical systems. As such, modeling these effects is essential for accurately simulating CR-SOA-based photonic devices, especially when operating under intense modulation and short pulse conditions. The resulting set of coupled differential equations captures the temporal dynamics of carrier densities, gain saturation, and phase modulation, forming the core framework for evaluating the CR-SOA’s suitability in all-optical logic and encryption schemes. The governing equations are provided as follows [11,12,13,14,15]:

(2) $$\frac{dh_{AR}(t)}{dt}=\frac{h_{CR}(t)-h_{AR}(t)}{\tau_{t}(1+\eta )}+\frac{\eta h_{0}}{\tau_{c}(1+\eta )}-\frac{h_{AR}(t)}{\tau_{c}}-(exp[h_{AR}(t)+h_{CH}(t)+h_{SHB}(t)]-1)\frac{P_{in,CR-SOA}(t)}{E_{sat}}$$

(3) $$\frac{dh_{CR}(t)}{dt}=-\frac{\eta (h_{CR}(t)-h_{AR}(t)}{\tau_{t}(1+\eta )}+\frac{h_{0}-h_{CR}(t)}{\tau_{c}(1+\eta )}-\frac{h_{CR}(t)}{\tau_{c}}$$

(4) $$\frac{dh_{CH}(t)}{dt}=-\frac{h_{CH}(t)}{\tau_{CH}}-\frac{\varepsilon_{CH}}{\tau_{CH}}(exp[h_{AR}(t)+h_{CH}(t)+h_{SHB}(t)]-1)P_{in,CR-SOA}(t)$$

(5) $$\frac{dh_{SHB}(t)}{dt}=-\frac{h_{SHB}(t)}{\tau_{SHB}}-\frac{\varepsilon_{SHB}}{\tau_{SHB}}(exp[h_{AR}(t)+h_{CH}(t)+h_{SHB}(t)]-1)P_{in,CR-SOA}(t)-\frac{dh_{AR}(t)}{dt}-\frac{dh_{CH}(t)}{dt}$$

where

- τ_t_ denotes the carrier transition lifetime from the CR to the AR, which governs the carrier transfer dynamics.
- τ_c_ represents the carrier recombination lifetime, dictating how quickly carriers recombine in AR.
- η is the population inversion factor, defined as the ratio of carrier densities in the AR and CR, i.e., η = *N_AR_*/*N_CR_*, indicating the efficiency of carrier injection into the gain medium.
- τ_CH_ is the thermal relaxation time associated with CH, which influences the speed of thermal carrier redistribution.
- τ_SHB_ denotes the relaxation time related to SHB, capturing the dynamics of carrier depletion at specific spectral components.
- ε_CH_ and ε_SHB_ are the nonlinear gain suppression coefficients arising from CH and SHB effects, respectively, reflecting the extent to which each mechanism depletes the available optical gain.

The total gain of the CR-SOA is thus a combined result of the gain contributions from the active region, modified by the suppressive impacts of CH and SHB. This comprehensive gain formulation is critical for modeling the amplifier’s performance under high-speed, high-intensity optical modulation.

(6) $$G_{CR-SOA_{i}}(t)=exp[h_{AR}(t)+h_{CH}(t)+h_{SHB}(t)],\;i=1,2,\ldots \ldots \ldots$$

The unsaturated power gain, G_0_, is given by:

(7) $$G_{0}=exp[h_{0}]=\Gamma \alpha \left(\frac{I\tau_{c}}{eV}-N_{tr}\right)L$$

where

- Γ is the optical confinement factor, representing the fraction of the optical mode that overlaps with the gain region, thereby influencing the efficiency of light amplification.
- α refers to the differential gain, a measure of how sensitively the optical gain responds to changes in carrier density.
- N_tr_ is the transparency carrier density, the minimum carrier concentration required to achieve zero net optical gain.
- I is the injection current, supplying the carriers needed for stimulated emission in the active region.
- e denotes the elementary charge of an electron, a fundamental constant used in calculating carrier densities.
- V = wdL represents the active layer volume, where w is the width, d is the thickness, and L is the length of the active region—this volume directly affects the carrier density and gain saturation characteristics.

The saturation energy (E_sat_) is a critical parameter that defines the energy threshold beyond which the gain of the CR-SOA begins to saturate. It quantifies the maximum optical input energy the device can efficiently amplify before nonlinear effects, such as gain compression and signal distortion, degrade performance. E_sat_ is calculated using the following expression:

(8) $$E_{sat}=P_{sat}\tau_{c}=\frac{wdh\upsilon }{\Gamma \alpha }$$

where

- P_sat_: Saturation power
- h: Planck’s constant
- υ: Signal frequency

For the numerical simulations, the input optical power pulses P_in, CR-SOA_(t) are modeled as return-to-zero (RZ) Gaussian-shaped pulses embedded within a pseudorandom binary sequence (PRBS) of length N. Each pulse is characterized by an energy E_0_, a full width at half-maximum (FWHM) duration τ_FWHM_, and a bit period T. This modeling approach accurately captures the temporal shape and data pattern of realistic high-speed optical signals used in communication systems. The PRBS pattern ensures a statistically representative sequence of logical ‘0’ and ‘1’ bits, essential for evaluating device performance under varied signal conditions. This pulse representation is mathematically described by the following equation:

(9) $$P_{A,B}(t)\equiv P_{in,CR-SOA}(t)=\sum_{n=1}^{N}a_{n(A,B)}\frac{2\sqrt{\ln[2}]E_{0}}{\sqrt{\pi }\tau_{FWHM}}\exp\left[-\frac{4\ln[2]{\left(t-nT\right)}^{2}}{\tau_{FWHM}^{2}}\right]$$

In this expression, a_n(A,B)_ denotes the PRBS data streams corresponding to the input signals A and B, where each bit can assume a logical value of either ‘0’ or ‘1’.

The induced phase shift in the CR-SOA is described by:

(10) $$\Phi_{CR-SOA_{i}}(t)=-0.5\left(\alpha h_{AR}(t)+\alpha_{CH}h_{CH}(t)\right),\;i=1,2,\ldots \ldots \ldots$$

where α represents the conventional linewidth enhancement factor (α-factor), and α_CH_ corresponds to the linewidth enhancement factor associated with CH. The contribution of SHB to the phase shift is considered negligible, with α_SHB_ ≈ 0 [15,18,21].

For numerical simulation of the encryption operation, the optical input powers applied to CR-SOA1 and CR-SOA2 within CR-SOA-MZI1 are mathematically modeled as follows (see Figure 3):

(11) $$P_{in,CR-SOA_{1}}(t)=P_{Data}(t)+0.5P_{CW}$$

(12) $$P_{in,CR-SOA_{2}}(t)=P_{Key}(t)+0.5P_{CW}$$

Subsequently, the output power corresponding to the encryption operation in CR-SOA-MZI1 is given by [11]:

(13) $$P_{Encryption}(t)=0.25P_{CW}\left(G_{CR-SOA_{1}}(t)+G_{CR-SOA_{2}}(t)-2\sqrt{G_{CR-SOA_{1}}(t)G_{CR-SOA_{2}}(t)}cos[\Phi_{CR-SOA_{1}}(t)-\Phi_{CR-SOA_{2}}(t)]\right)$$

To carry out the decryption process, the optical input powers injected into CR-SOA3 and CR-SOA4 within CR-SOA-MZI2 are defined as follows:

(14) $$P_{in,CR-SOA_{3}}(t)=P_{Encryption}(t)+0.5P_{CW}$$

(15) $$P_{in,CR-SOA_{4}}(t)=P_{Key}(t)+0.5P_{CW}$$

Consequently, the output power representing the decryption operation, retrieved from the decryption process in CR-SOA-MZI2, is given by:

(16) $$P_{Decryption}(t)=0.25P_{CW}\left(G_{CR-SOA_{3}}(t)+G_{CR-SOA_{4}}(t)-2\sqrt{G_{CR-SOA_{3}}(t)G_{CR-SOA_{4}}(t)}cos[\Phi_{CR-SOA_{3}}(t)-\Phi_{CR-SOA_{4}}(t)]\right)$$

## 4. Results and Discussion

**Figure 4:**
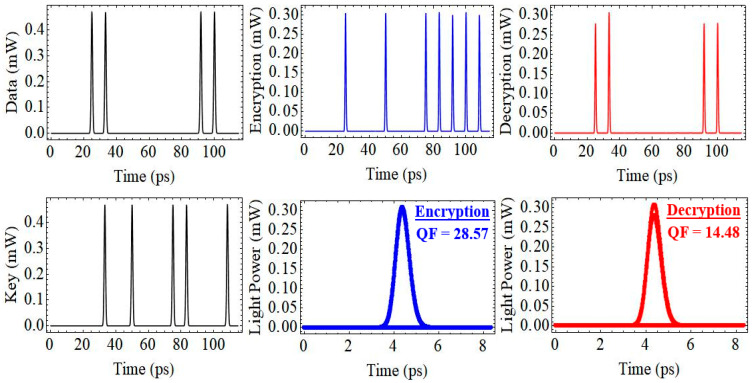
Temporal waveforms and eye diagrams for the CR-SOA-MZI-based encryption/decryption at 120 Gb/s. The inputs are the Data and Key signals. The middle panels show the encrypted output and its eye diagram (QF = 28.57), while the right panels show the decrypted output and corresponding eye diagram (QF = 14.48), demonstrating clear signal recovery and high system integrity.

The performance of the proposed CR-SOA-MZI-based all-optical encryption and decryption system was evaluated through comprehensive numerical simulations using Wolfram Mathematica^®^ at a data rate of 120 Gb/s. The architecture consists of two cascaded XOR logic gates realized through CR-SOAs embedded in MZIs (CR-SOA-MZI1 and CR-SOA-MZI2). The system receives two input signals: a pseudorandom optical data stream (Signal A) and a secure optical key (Signal B). The encryption process is performed in CR-SOA-MZI1 using the XOR operation with key B, while decryption is achieved in CR-SOA-MZI2 using the same key, based on the logical identity (A ⊕ B) ⊕ B. Figure 4 illustrates the temporal waveforms of the data, key, encrypted, and decrypted signals. The plots shown in the bottom middle and bottom right panels represent single-bit output waveforms (logical ‘1’) from the encryption and decryption stages, respectively. While these were previously referred to as “eye diagrams,” we clarify that they are not conventional eye diagrams involving multiple overlapping bits. Instead, they were extracted to highlight pulse sharpness, timing precision, and signal amplitude under high-speed operation. We recognize the value of including standard eye diagrams to illustrate jitter, noise, and inter-symbol interference, and we plan to present such plots in future work. The QF of the encrypted signal (bottom middle, blue) is 28.57, demonstrating a clean, high-contrast signal with minimal distortion. In comparison, the decrypted signal (bottom right, red) yields a QF of 14.48, reflecting some amplitude degradation and broader temporal features resulting from cumulative pattern-dependent effects and dynamic gain variations in the CR-SOAs. These effects are more pronounced after the second XOR stage, contributing to the observed reduction in QF. Nonetheless, both signals maintain strong temporal integrity, validating the logical accuracy and high fidelity of the encryption and decryption processes. The QF values reflect the effectiveness of the CR-SOA’s fast gain recovery and carrier dynamics in maintaining logic stability under ultrafast modulation conditions. Additionally, the system exhibits resilience to amplified spontaneous emission (ASE) noise, which can be attributed to the intrinsic noise suppression capabilities of the CR-SOA-MZI configuration. The fast carrier recovery in the CR-SOAs rapidly mitigates noise-induced gain fluctuations, while the nonlinear XOR logic operation within the MZI enhances signal contrast, effectively filtering out ASE noise contributions. Consequently, these characteristics ensure stable operation and high signal fidelity, confirming the system’s potential for secure and scalable high-speed optical communication.

**Figure 5:**
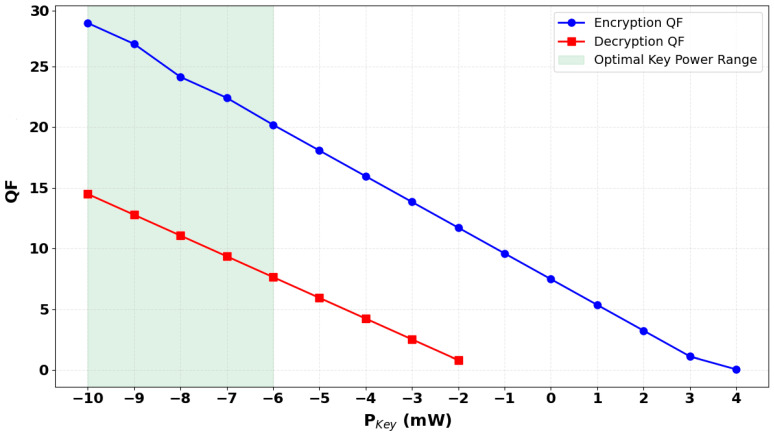
QF of encrypted and decrypted signals versus key input power (P_Key_), showing optimal performance at low key power and degradation at higher levels due to nonlinear effects.

To further evaluate the robustness and sensitivity of the proposed CR-SOA-MZI-based all-optical security system, the effect of input key power on the quality factors (QFs) of both the encrypted and decrypted signals is thoroughly analyzed. The key input plays a pivotal role in the encryption and decryption processes, serving not only as a logical operand in the XOR operation but also as a dynamic control signal that governs the nonlinear interference characteristics within the CR-SOA-assisted MZIs. Figure 5 presents the variation in QF values for both encryption and decryption outputs as a function of input key power (P_Key_), ranging from −10 dBm to +4 dBm. The results indicate a clear performance degradation trend in both encryption and decryption QFs as P_Key_ increases. At a P_Key_ of −10 dBm, the encryption and decryption processes demonstrate excellent signal quality, with QFs of 28.57 and 14.48, respectively. These values reflect clean, well-separated logic levels with minimal inter-symbol interference, confirming that the system operates optimally under low key power conditions. As P_Key_ increases from −9 dBm to −5 dBm, the QFs of both encrypted and decrypted signals decline gradually. For instance, at −6 dBm, the encryption QF drops to 20.19, and the decryption QF reduces to 7.64, indicating growing distortion in the logical transitions. This degradation was determined through simulation outputs that revealed temporal broadening, waveform distortion, and diminished contrast at higher input powers. These signal impairments correlated with known nonlinear optical effects—including enhanced cross-phase modulation (XPM) and gain saturation—captured in the dynamic model of the CR-SOA. Specifically, the simulations incorporated carrier rate equations and field propagation dynamics, which reflect the impact of increased input power on gain recovery, phase shift, and interferometric balance. At higher P_Key_ levels, these effects become more prominent, reducing the effectiveness of the XOR logic. Beyond −4 dBm, the performance degrades rapidly. The decryption QF falls below the acceptable threshold (QF < 3) by approximately −3 dBm, suggesting that the recovered signal becomes highly distorted and difficult to interpret. At −2 dBm, the decryption QF drops to 0.8, while encryption still retains 11.71, demonstrating that encryption remains relatively more robust but also begins to lose fidelity. Beyond 0 dBm, the decryption signal fails almost entirely, with QFs nearing zero by +4 dBm, indicating that the decrypted signal is no longer distinguishable due to excessive nonlinear interference and phase mismatch in the CR-SOA-MZI2 structure. This behavior highlights a critical trade-off: while a certain level of optical power is necessary for maintaining gain and facilitating nonlinear interaction in CR-SOAs, excessive key power introduces detrimental effects, such as carrier depletion, XGM, and XPM, that hinder the proper operation of the XOR gates. The encrypted output remains functional longer due to its position earlier in the cascade (CR-SOA-MZI1), whereas the decryption gate (CR-SOA-MZI2) is more vulnerable to upstream distortions and cumulative ASE noise. In addition to performance sensitivity, the overall power efficiency of the proposed architecture has been estimated based on simulation parameters. The system operates optimally when the key input power lies within the range of −10 dBm to −6 dBm, where both encrypted and decrypted signals retain high QF values. Under these conditions, and accounting for component losses and CR-SOA gain, the net optical power efficiency is estimated to be 15–20%. This range represents a trade-off between ensuring sufficient nonlinear interaction and avoiding excessive phase distortion or gain saturation. The results suggest that proper key power tuning not only ensures secure encryption but also supports energy-efficient operation of the system. These results underscore the importance of carefully tuning P_Key_ to maintain system integrity. The optimal key power window lies around −10 dBm to −6 dBm, within which both encrypted and decrypted outputs retain acceptable QFs for reliable transmission and recovery. Outside this range, especially for positive key powers, the system becomes increasingly susceptible to degradation. Furthermore, the simulations affirm that CR-SOAs offer strong potential for high-speed secure operations, provided that nonlinearities are properly managed. The performance sensitivity to key power also reinforces the value of power-aware encryption architectures, where the intensity of the key serves as a secondary security parameter—any deviation from the optimal power range can render the decryption process ineffective, thereby enhancing the system’s resilience against unauthorized access.

**Figure 6:**
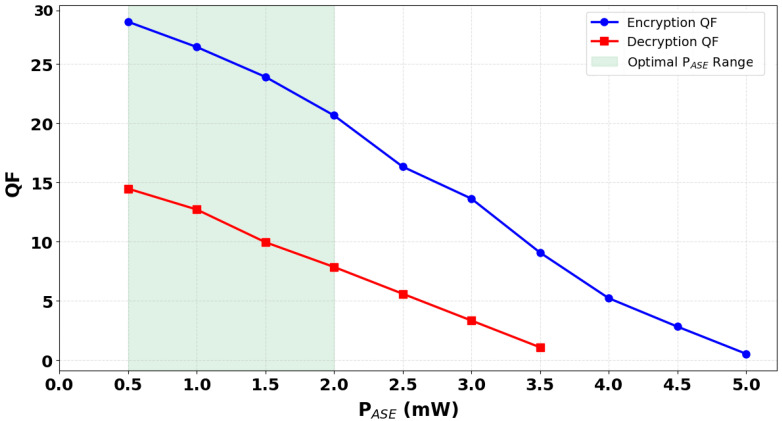
QF of encrypted and decrypted signals versus amplified spontaneous emission power (P_ASE_), showing degradation at higher ASE levels due to increased noise impact.

In this study, we numerically incorporated amplified spontaneous emission (ASE) noise into the output power equations for both encryption and decryption, specifically Equations (13) and (16), to realistically assess the performance of our 120 Gb/s all-optical system based on CR-SOAs-MZIs. ASE is a fundamental noise mechanism originating from spontaneous carrier recombination events in the SOA’s gain medium, which emits incoherent photons that accumulate along with the amplified signal. Unlike stimulated emission, ASE contributes broadband noise that degrades the signal’s coherence and reduces the signal-to-noise ratio, particularly detrimental in high-speed photonic logic systems. To capture this degradation, ASE power [18] was systematically varied and added numerically to the output power calculations. As shown in Figure 6, the QFs for both encryption and decryption decrease sharply with increasing ASE power. Specifically, when the ASE power increases from 0.5 mW to 5 mW, the encryption QF drops from 28.57 to 0.54, while the decryption QF declines from 14.48 to nearly zero. This inverse relationship illustrates the detrimental impact of ASE on system performance. Interestingly, at each level of ASE power, the QF for encryption is consistently higher than that for decryption. This behavior suggests a differing sensitivity of the two operations to ASE noise, likely arising from asymmetries in gain saturation, nonlinear phase shift accumulation, or interference contrast between the arms of the MZI during signal recombination. By numerically including ASE in the model, we achieve a more realistic representation of system behavior under noisy conditions, moving beyond idealized assumptions. This approach enables accurate prediction of performance limits and helps define acceptable operating windows for SOA biasing and input power levels. The results emphasize that even moderate increases in ASE can significantly impair logic operation, especially at ultrafast speeds such as 120 Gb/s, where signal degradation leads directly to reduced encryption fidelity and unreliable decryption. Therefore, mitigating ASE through optimized SOA design, use of narrowband optical filters, and controlled operating parameters is critical to maintaining high quality factors and ensuring the robustness and security of the all-optical logic gate system.

## 5. Comparative Performance Evaluation

The proposed all-optical encryption and decryption system employing CR-SOAs-MZIs operating at 120 Gb/s marks a significant leap forward compared to prior SOA-based architectures in both data rate and signal integrity, as evidenced by the results in Table 3. Initial experimental demonstrations, such as those by Son et al. [1] and Jung et al. [2], utilized conventional SOAs and were limited to data rates of 10 Gb/s and 2.5 Gb/s, respectively, achieving modest quality factors (QF ≈ 5.4) and extinction ratios (ER ≈ 7.0 dB). These limitations were largely attributed to the slow carrier recovery time of traditional SOAs, which introduces pattern-dependent distortions and restricts scalability to higher bit rates. To address this, Yang et al. [3] proposed an SOA-based encryption/decryption scheme leveraging four-wave mixing (FWM) for non-return-to-zero differential phase-shift keying (NRZ-DPSK) signals at 10 Gb/s, achieving ERs of 9.0 dB and 9.3 dB. Despite the added benefit of wavelength conversion, FWM-based systems are highly sensitive to phase mismatch and suffer from design complexity. Simulation-based designs have pushed the performance boundaries further. Agarwal and Chaurasia [4] presented a 40 Gb/s system using copropagating pulses and CW inputs in SOA-MZI configurations, reporting a QF of 13.50. More recently, Singhal et al. [5] developed a 250 Gb/s encryption/decryption circuit using SOAs and demonstrated strong simulated performance with QF = 19.65 dB and ER = 21.2 dB. Zhang et al. [6] introduced a two-photon absorption (TPA) mechanism in SOAs at 250 Gb/s, though the QF was limited to 7.20 due to nonlinear impairments and sensitivity to input power. In a separate work, Li et al. [7] utilized quantum dot SOAs (QD-SOAs) for high-speed encryption, achieving a QF of 8.20, but practical deployment remains constrained by fabrication and integration challenges associated with QD structures. A notable improvement came from Bosu and Bhattacharjee [8], who proposed encryption and decryption circuits using reflective SOAs (RSOAs) at 100 Gb/s. Their simulated results revealed excellent performance with QFs of 25.28 dB and 18.27 dB, and contrast ratios (CRs) of 41.12 dB and 29.52 dB. However, RSOAs require tight control over optical feedback and cavity design, complicating integration into scalable systems. In comparison, the CR-SOA-based encryption and decryption system presented in this work operates at 120 Gb/s and achieves superior simulated QFs of 28.57 and 14.48 under varying input key powers. This high-speed performance is primarily attributed to the unique carrier reservoir structure, which enables faster carrier recovery, minimizes gain saturation, and ensures robust logic operation under realistic conditions. Unlike previously reported systems that rely heavily on idealized simulations or complex nonlinear effects, the proposed CR-SOA-MZI architecture strikes an optimal balance between speed, integration simplicity, and performance. These findings establish this work as a new benchmark for future secure, ultrafast, and energy-efficient all-optical data processing systems suitable for deployment in high-capacity photonic communication infrastructures.

## 6. Proposed Experimental Framework

**Figure 7:**
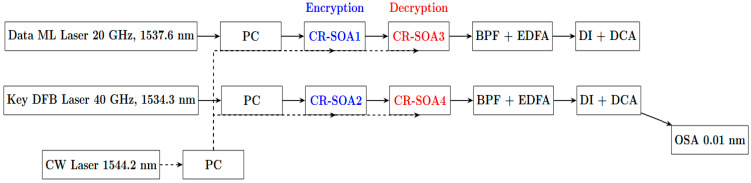
Experimental setup for all-optical encryption and decryption at 120 Gb/s using CR-SOAs within MZIs. It includes synchronized data and key lasers, a CW pump laser for nonlinear enhancement, polarization controllers (PCs), cascaded CR-SOA-MZI XOR gates, optical filters (BPFs), EDFAs, delay interferometers (DIs), and a digital communications analyzer (DCA) for system evaluation.

To provide a practical perspective on implementing the all-optical encryption and decryption scheme demonstrated by our simulations, we outline a conceptual experimental framework aimed at operation at 120 Gb/s (Figure 7). This framework employs CR-SOAs integrated within MZIs to exploit their fast nonlinear dynamics for ultrafast XOR-based encryption and decryption operations. The input signals are RZ Gaussian pulses with approximately 3.5 ps temporal widths, generated by two synchronized lasers: a rational harmonic mode-locked fiber laser at 20 GHz produces the data stream (signal A) centered at 1537.6 nm, while a gain-switched distributed feedback laser at 40 GHz provides the encryption key stream (signal B) centered at 1534.3 nm. Both lasers are phase-locked via a shared 10 GHz radio frequency (RF) synthesizer to ensure precise temporal alignment, crucial for pulse interleaving and time-division multiplexing, enabling an effective aggregate data rate of 120 Gb/s. A CW laser at 1544.2 nm, operating with an output power carefully controlled near 5.6 dBm, is injected as a pump beam to enhance XGM within the CR-SOAs, which is the essential nonlinear effect facilitating ultrafast all-optical logic required for encryption and decryption. Polarization controllers preceding each CR-SOA-MZI input port guarantee all injected signals—data, key, and CW—are co-polarized to maximize nonlinear interaction efficiency and reduce polarization-induced phase errors. The CR-SOAs used are commercial devices approximately 1 mm in length, providing over 25 dB small-signal gain and optimized carrier reservoir dynamics, biased at approximately 300 mA and temperature stabilized at 25 °C to support fast gain recovery needed for 120 Gb/s operation. Four CR-SOA-MZIs are arranged to perform fundamental logic functions integral to encryption and decryption: two MZIs implement complementary XOR components for encryption by combining data and key signals, while subsequent stages enable decryption by applying the key stream again via XOR operation on the encrypted data. Input signals are split with 3 dB optical couplers and routed through wavelength selective couplers and finely tuned optical delay lines to synchronize pulses temporally inside the interferometers, optimizing interference contrast and logic accuracy. Post-SOA outputs pass through narrowband optical band-pass filters (~0.5 nm bandwidth) to select desired wavelength channels and suppress ASE noise and four-wave mixing idlers, followed by amplification with erbium-doped fiber amplifiers (EDFAs) to ensure sufficient power (~5 to 10 dBm) for accurate detection. Differential delay interferometers convert the phase-encoded RZ Gaussian signals into intensity-modulated formats for real-time capture using a high-speed digital communications analyzer (DCA), which evaluates system performance metrics such as eye diagrams, extinction ratios, and quality factors. An optical spectrum analyzer with 0.01 nm resolution monitors the spectral purity and wavelength stability throughout the setup. Input power levels are precisely adjusted, with data and key signals maintained between 2.3 and 3.7 dBm and the CW pump fixed near 5.6 dBm, balancing nonlinear interaction efficiency against noise and distortion. This proposed experimental framework serves as a practical guide to realizing the CR-SOA-MZI system and demonstrates the feasibility of achieving high-quality, ultrafast all-optical encryption and decryption at the record data rate of 120 Gb/s. Experimental verification based on this framework is planned as a future extension of the current work.

## 7. Conclusions

This work presents a high-speed all-optical encryption and decryption system operating at 120 Gb/s, utilizing CR-SOAs embedded in MZIs. By cascading two XOR logic gates, the system performs both encryption and decryption using a shared optical key, ensuring secure and ultrafast data processing through phase-sensitive interference. Numerical simulations using Wolfram Mathematica^®^ confirm excellent signal integrity, with QFs of 28.57 and 14.48 for the encrypted and decrypted signals, respectively. The system demonstrates high resilience under low-key power and moderate ASE noise, with performance degrading only beyond specific thresholds. This analysis reveals the critical importance of input key power optimization, as excessive nonlinearities can compromise logic fidelity, particularly in the decryption stage. Compared to previous SOA- and QD-SOA-based systems, the CR-SOA-MZI architecture achieves superior performance in terms of speed, integration simplicity, and robustness. The fast carrier recovery and reduced pattern dependence of CR-SOAs enable scalable, low-latency, and noise-tolerant operation, positioning this approach as a strong candidate for next-generation secure photonic networks. In summary, this study establishes a solid foundation for the development of energy-efficient, ultrafast all-optical security systems. Future efforts will focus on experimental validation and system-level integration to extend the applicability of this architecture to real-world optical communication infrastructures.

## Figures and Tables

**Table 1:** XOR-based encryption and decryption logic using CR-SOA-MZIs.

Inputs		Outputs	
		Encryption	Decryption
A	B	A ⊕ B	(A ⊕ B) ⊕ B = A
0	0	0	0
0	1	0	0
1	0	1	1
1	1	1	1

**Table 2:** The default numerical values and parameters applied in this study [1,2,3,4,5,6,7,8,9,10,11,12,13,14,15,16,17,18,19,20,21].

Symbol	Definition	Value	Unit
E_0_	Pulse energy	0.5	pJ
τ_FWHM_	Pulse width	1	ps
T	Bit period	8.33	ps
N	PRBS length	127	-
λ_Data_	Wavelength of data	1537.6	nm
λ_Key_	Wavelength of key	1534.3	nm
λ_CW_	Wavelength of CW	1544.2	nm
I	Injection current	300	mA
P_sat_	Saturation power	30	mW
τ_c_	Carrier lifetime	200	ps
τ_t_	Transition lifetime from CR to AR	5	ps
η	Population inversion factor	0.3	-
α	α-factor	5	-
α_CH_	Linewidth enhancement factor due to CH	1	-
α_SHB_	Linewidth enhancement factor due to SHB	0	-
ε_CH_	Nonlinear gain suppression factor due to CH	0.2	W^−1^
ε_SHB_	Nonlinear gain suppression factor due to SHB	0.2	W^−1^
τ_CH_	Temperature relaxation rate	0.3	ps
τ_SHB_	Carrier-carrier scattering rate	0.1	ps
Γ	Optical confinement factor	0.3	-
α	Differential gain	10^−20^	m^2^
N_tr_	Transparency carrier density	10^24^	m^−3^
L	Length of AR	500	μm
d	Thickness of AR	0.3	μm
w	Width of AR	3	μm
G_0_	Unsaturated power gain	30	dB
ω_0_	Central optical frequency	193.55	THz
N_SP_	Spontaneous emission factor	2	-
B_0_	Optical bandwidth	2	nm

**Table 3:** Comparison of all-optical encryption/decryption schemes based on different SOA architectures, showing data rate, methodology, and performance metrics. The proposed CR-SOA system operates at 120 Gb/s with high QF.

Ref.	Operations	Scheme	Data Rate (Gb/s)	Methodology	Metric
[1,2]	Encryption/Decryption	SOA	10	Sim.	QF = 5.4
	Encryption/Decryption	SOA	2.5	Exp.	ER = 7.0 dB and 5.5 dB
[3]	Encryption/Decryption	SOA-FWM	10	Exp.	ER = 9.0 dB and 9.3 dB
[4]	Encryption/Decryption	SOA	40	Sim.	QF = 13.50
[5]	Encryption/Decryption	SOA	250	Sim.	QF = 19.65 dB
					ER = 21.20 dB
[6]	Encryption/Decryption	SOA-TPA	250	Sim.	QF = 7.20
[7]		QD-SOA	250	Sim.	QF = 8.20
[8]	Encryption/Decryption	RSOA	100	Sim.	QF = 25.28 dB and 18.27 Db
					CR = 41.12 Db and 29.52 Db
This work	Encryption/Decryption	CR-SOA	120	Sim.	QF = 28.57 and 14.48

## Data Availability

Data are contained within the article.

## Abbreviations

SOA	Semiconductor Optical Amplifier
CR-SOA	Carrier Reservoir Semiconductor Optical Amplifier
QD-SOA	Quantum-Dot Semiconductor Optical Amplifier
RSOA	Reflective Semiconductor Optical Amplifier
MZI	Mach–Zehnder Interferometer
XOR	Exclusive-OR
QF	Quality Factor
BER	Bit Error Rate
ER	Extinction Ratio
XGM	Cross-Gain Modulation
XPM	Cross-Phase Modulation
TPA	Two-Photon Absorption
ASE	Amplified Spontaneous Emission
CR	Carrier Reservoir
AR	Active Region
CW	Continues Wave
OC	Optical Coupler
WSC	Wavelength-Selective Coupler
OBPF	Optical Bandpass Filter
RZ	Return-to-Zero
FWHM	Full Wave at Half Maximum
EDFA	Erbium-Doped Fiber Amplifier
DCA	Digital Communications Analyzer
DI	Delayed interferometer
PC	Polarization Controller
